# Corrigendum: Contribution and distribution of inorganic ions and organic compounds to the osmotic adjustment in *Halostachys caspica* response to salt stress

**DOI:** 10.1038/srep15867

**Published:** 2015-12-14

**Authors:** Youling Zeng, Ling Li, Ruirui Yang, Xiaoya Yi, Baohong Zhang

Scientific Reports 5: Article number: 1363910.1038/srep13639; published online: 09092015; updated: 12142015

In the Supplementary Information originally published with this Article, there was a typographical error in Table S1: ‘CO_3_^–^’ now reads ‘CO_3_^2–^’. This error has been corrected in the Supplementary Information that now accompanies the Article.

In addition, a proof of the Article was inadvertently published as Supplementary Information. This has now been removed.

