# Supplementary material for: Contribution and distribution of inorganic ions and organic compounds to the osmotic adjustment in Halostachys caspica response to salt stress
Source: Sci Rep. 2015 Sep 9;5:13639. doi: 10.1038/srep13639 (PMC4563356; doi:10.1038/srep13639)
Supplement: Supplementary Information [file srep13639-s1.doc]

**Contribution and distribution of inorganic ions and organic compounds to the osmotic adjustment in** ***Halostachys caspica* response to salt stress**

Youling Zeng ^1, *^, Ling Li ^1^, Ruirui Yang ^1^, Xiaoya Yi ^1^ & Baohong Zhang ^2^

^1^ Xinjiang Key Laboratory of Biological Resources and Genetic Engineering, College of Life Science and Technology, Xinjiang University, Urumqi 830046, China, ^2^ Department of Biology, East Carolina University, Greenville, NC 27858, USA

* To whom correspondence should be addressed. E-mail: zengyouling@xju.edu.cn

**Abstract:**

The mechanism by which plants cope with salt stress remains poorly understood. The goal of this study is to systematically investigate the contribution and distribution of inorganic ions and organic compounds to the osmotic adjustment (OA) in the halophyte species *Halostachys caspica*. The results indicate that 100-200mM NaCl is optimal for plant growth; the water content and degree of succulence of the assimilating branches are higher in this treatment range than that in other treatments; parenchyma cells are more numerous with 100mM NaCl treatment than they are in control. Inorganic ions (mainly Na+ and Cl-) may play a more important role than organic compounds in NaCl-induced OA and are the primary contributors among organic compounds in OA in the *H. caspica* assimilating bran. The inorganic ions and organic solutes display a tissue-dependent distribution. Na^+^ and Cl^−^ are accumulated in the reproductive organs and within assimilating branches with higher succulenca mechanism for protecting plant growth by way of salt ion dilution and organ abscission. Additionally, osmotic adjustmentOA vulation of organic substances in the roots and reprorowth and development. This finding provides additional evidence for plant tolerance to salinity stress which can be used for breeding new cultivars for stress tolerance.

**Keywords:** *Halostachys caspica*, succulence, osmotic adjustment, inorganic ions, organic compounds, contribution, tissue distribution, salt stress

Soil salinization is a major abiotic environmental factor that limits land utilization efficiency in arid and semi-arid regions worldwide and reduces the yield of a wide variety of crops ^1^. Considering the combination of a high evapotranspiration rate and poor quality water irrigation, salinity stress adversely impacts plant metabolism through ion toxicity, osmotic stress and oxidative stress. The ability of certain plants to survive and maintain adequate growth under high salinity conditions is referred to as salt tolerance; this variable trait is dependent on many factors including the plant species. For example, halophytic plants can survive and even grow well under high salt concentrations in the rhizosphere ^2, 3^. Nevertheless, the tissues and organs of halophytes are also susceptible to extreme salt conditions in terms of morphology and physiology. In recent decades, the distribution, exploitation ^4^ and physiology of halophyte salt tolerance ^5, 6^ have been extensively studied. Zhao described three different modes of salt tolerance response observed in terrestrial plants that allow them to overcome salt ion toxicity: salt dilution, salt secretion and salt exclusion ^7^. The response to osmotic stress primarily involves osmotic adjustment (OA), which refers to a reduction of osmotic potential in response to a net accumulation of solutes resulting from water deficiency or salinity. OA is critical for maintaining cell turgor, which enables the maintenance of plant metabolic activity and in turn plant growth and productivity ^8, 9^. It is generally recognized that osmotic substances can reduce the effects of ion toxicity and form various complexes with proteins or bind to their surfaces via a hydrophilic protecting osmotic function ^10^. Greenway previously described the synthesis of compatible osmotic substances as a metabolic response adaptation to salt stress ^11^. In contrast to glycophytes, halophytes have adapted various physiological and biochemical strategies to cope with extreme salt conditions ^12^. These include tissue succulence and ion compartmentation that involve the elimination or accumulation of excess saline ions (Na^+^) in the vacuoles. Plants also synthesize proline, glycine, betaine, soluble sugars and other osmolytes to promote osmotic balance at the cellular level ^13-16^. As one example, plants belonging to the Chenopodiaceae family use the inorganic ions Na^+^ and Cl^−^ as osmotic agents ^4^.

Although halophytes are known to have evolved several adaptive mechanisms to cope with the presence of high level of salts in their natural environments ^7, 17-19^, these mechanisms remain poorly understood. The contribution of inorganic ions and organic solutes to OA continues to be disputed, and the strategies used for stress tolerance are known to vary not only by species but also by genotype ^20^. For instance, the distribution of osmotic substances varies among dicotyledonous halophytes, monocotyledonous halophytes and true halophytic plants ^21^.

The halophyte *Halostachys caspica* (Bieb.) C.A.Mey is a salt-diluted short shrub belonging to the Chenopodiaceae family. It exhibits high salt tolerance and grows naturally on the saline-alkali soil of semi-desert regions in Central Asia ^22^. The unique morphological structures of *H. caspica* are its assimilating branches formed by retrogressed leaves, which are capable of hyperaccumulating Na^+^ and Cl^−^. At present, little is known about the role of OA in the salt tolerance response of *H. caspica* at the physiological level, despite the recognition of OA as an important physiological adaptation of halophytic species with high salt tolerance.

The aims of the present study were to assess the contribution of inorganic ions and organic compounds to the OA of *H. caspica* plants in response to salt stress, to determine the contribution and distribution of these molecules, and to identify the optimal salt concentration for the growth and development of *H. caspica*. It is very important to clarify these physiological characteristics; it also is a good reference for fully exploring the salt tolerance mechanisms on a physiological level.

**Results**

**Optimal salt concentrations for plant growth and development**

The *H. caspica* plants were grown for four weeks in the greenhouse. ith e them flattened fully, of the slicento the , A range of NaCl concentrations (0, 100, 200, 300, 400, 500 and 600 mM) were used for the experimental treatments. Plants treated with 100 or 200 mM NaCl grew very well. However, plants treated with 300 to 600 mM NaCl exhibited growth inhibition, including yellowed assimilating branches and reduced plant size (Fig. 1). Nevertheless, these visual observations clearly indicated the high tolerance of *H. caspica* to NaCl salinity. With increasing salt stress up to 200 mM NaCl, the *H. caspica* plants also exhibited a stable water content (WC). As the NaCl concentration increased from 400 to 600 mM, the WC decreased significantly in the assimilating branches compared to the control (P<0.01). With the treatment of 600 mM NaCl, the WC remained as high as 88.02% (Fig. 2a). The succulence of *H. caspica* assimilating branches, measured as the FW/DW ([fresh weight]/[dry weight]) ratio, increased significantly as the NaCl treatment increased from 0 to 200 mM, but decreased prominently thereafter (Fig. 2b). The histological analysis of the anatomical structure of *H. caspica* assimilating branches (Fig. 3) revealed that the number and size of parenchyma cells in this part of the plant increased with 100 mM NaCl treatment. Qi et el. (2005) similarly reported that the degree of succulence of the halophyte *Suaeda salsa* is highly correlated with salinity levels and that maximum succulence is observed under saline conditions ^23^. Additionally, Radić et al. (2013) found that low salt concentrations induced leaf succulence and increased the relative WC of the leaves of *Centaurea ragusina* ^24^. In the present study, optimal plant growth was achieved with NaCl concentrations of 100 to 200 mM. The salt sensitivity of *H. caspica* was clearly demonstrated by the occurrence of toxicity symptoms that were visually observed in the assimilating branches as the NaCl concentration increased from 400 to 600 mM.

Salt stress was found to significantly affect the accumulation of Na^+^, which increased in the salt-stressed assimilating branches as the NaCl concentration increased. For instance, the accumulation of Na^+^ was 2.17 times greater in assimilating branches treated with 600 mM NaCl compared to the control (Fig. 4a).

A slight descending trend was observed for the accumulation of K^+^ in the salt-stressed shoots compared to the control (Fig. 4b), but none of the changes were significant among the different NaCl concentrations used (0–600 mM). At the highest NaCl treatment level (600 mM), the K^+^ concentration in assimilating branches was 68.29% of the concentration observed in the control. The accumulation of Ca^2+^ gradually decreased with increasing NaCl concentration, with the Ca^2+^ concentration observed at the highest salinity level approximately half (50.82%) of the Ca^2+^ concentration observed in the control (Fig. 4c). Due to the increased Na^+^ content in assimilating branches, the Na^+^/K^+^ ratio increased from 0 to 300 mM NaCl; however, no significant changes were observed beyond that concentrations (Fig. 4d). A similar tendency was observed for the Na^+^/Ca^2+^ ratio in treated *H. caspica* (Fig. 4e).

Betaine, proline (Pro) and total soluble sugars (TSS) were the three major organic solutes investigated, and the contents of all three solutes significantly increased in the assimilating branches with increasing NaCl levels (Fig. 5). When the plants were treated with 600 mM NaCl, the levels of betaine and Pro were increased by >16-folds and >136-folds, respectively.

**The contribution of inorganic ions and organic compounds to osmotic adjustment in response to salt stress**

The contribution of organic and inorganic solutes to OA in the assimilating branches of *H. caspica* is summarized in Table 1. Under 600 mM NaCl treatment, the Na^+^ and Cl^−^ ions made up 32.7% and 26.7%, respectively, of the total solutes contributing to OA. Even in the control, the contribution of Na^+^ and Cl^−^ was 45% and 31%, respectively. These findings indicate that Na^+^ and Cl^−^ play an important role in the OA of assimilating branches. Among the organic solutes, betaine was found to have the greatest contribution to OA in response to salt stress, accounting for 7–34% of the total solutes. In the tested NaCl range, the contributions of TSS and Pro were small; for example, the values for Pro were always below 1% of the total solutes. Although the total inorganic ion contribution decreased with increasing salt concentration, it nevertheless represented the main component at all concentrations. The mole fraction of inorganic ions ranged from 59%-90%, while that of organic solvents ranged from 10%-37%. Taken together, the above data indicate that inorganic ions and the organic solute betaine are major contributors to OA.

**Distribution of inorganic ions and organic solutes in different tissues and organs**

The content of inorganic ions was determined in various organs of mature plants. The highest levels of Na^+^ and Cl^−^ were observed in assimilating branches and reproductive organs, and these indexes were similarly high in the xylem of assimilating branches (ABX) and the xylem of reproductive organs (ROX). High Na^+^ and Cl^−^ levels were also observed in the roots. In contrast, K^+^ primarily accumulated in the reproductive organs, with lower accumulation observed in the roots, ABX, assimilating branches and ROX. Ca^2+^ accumulated mainly in the roots, which strongly contrasted the patterns observed in the above-ground organs. No particular distribution patterns were observed for SO_4_^2-^ and Mg^2+^ (Fig. 6).

Figure 7 shows that the distribution of inorganic ions in *H. caspica* seedlings was similar to that of the mature plants. A significantly higher accumulation of Na^+^, Cl^−^, SO4^2-^ and Mg^2+^ was observed in the shoots compared to the roots (P<0.001). In contrast, there was a greater accumulation of K^+^ and Ca^2+^ in the roots compared to the shoots (P<0.001) (Fig.7).

The difference in the Na^+^ partitioning in assimilating branches was greater than that of the Cl^−^ partitioning. Specifically, the Na^+^ content of the assimilating branches was 2250 mmol/kg DW, while the Na^+^ content of the roots was 580 mmol/kg DW.

The distribution of organic solutes in seedlings and mature *H. caspica* plants was also analyzed. Our results show that the accumulation of Pro, betaine and TSS was much higher in the reproductive organs of mature plants than in that in the assimilating branches (P<0.01) for samples collected from the saline-alkali soil. For the samples collected from relatively moderate saline-alkali soil, the betaine and Pro concentrations were both low, and no obvious differences were observed between the reproductive organs and assimilating branches. The concentrations of Pro and TSS in the reproductive organs of *H. caspica* collected from saline-alkali soil were greater than that in the corresponding concentrations of plants collected from low saline soil, and the values recorded for these two growth conditions were 1103.87 and 549.57 μg/g FW, respectively, for Pro and 242.89 and 37.96 mg/g FW, respectively, for TSS. Compared to the assimilating branches, the roots of seedlings from saline-alkali soil had a higher accumulation of organic solutes. This was particularly evident for the accumulation of TSS, whose levels in the roots were nearly 11.75-fold greater than those in the shoots (Fig. 8).

**Discussion**

Osmotic stress and ion toxicity are often associated with excessive salt concentration through as yet unknown mechanisms, ultimately resulting in reduced plant growth and development. Adaptive mechanisms at the cellular level, such as salt ion compartmentation and OA, are crucial for the survival and continued growth of salt-tolerant plants in high salinity environments ^25^. Salt-tolerant plants, halophytes in particular, accumulate large amounts of salt ions in their cells, with the vacuoles playing a key role in OA. Salt induces the accumulation of organic solutes compatible with biomolecular functions that underlie cellular protection and OA, especially in the cytoplasm and organelles ^26, 27^. Because ion homeostasis is essential for plant growth and development, the accumulation of excessive Na^+^ and Cl^−^ ions may disrupt this balance.

In the present study, plant growth was significantly inhibited by 400–600 mM NaCl treatments (Fig. 1). On the other hand, optimal plant growth and succulence were observed when the plants were treated with 100 or 200 mM NaCl (Figs. 1, 2 and 3). These findings suggest that *H. caspica* has an efficient system underlying OA under certain levels of NaCl stress. In general, reductions in WC represent a quick and economical approach for achieving OA in response to osmotic stress in plants ^28^. This phenomenon was indeed observed for *H. caspica* in the present investigation: decreased WC and/or succulence occurred at high salinity levels (400–600 mM NaCl) and likely resulted from disturbed osmotic homeostasis and the occurrence of dehydration due to excess Na^+^ accumulation (Fig. 2a).

Although the WC of *H. caspica* decreased significantly with increasing salt stress (Fig. 2a), a high WC was nevertheless maintained even under 600 mM NaCl (88.02%). Similar findings have been reported in other plants ^28-31^. Thus, the ability of *H. caspica* to retain water under severe salt stress may be a key characteristic of its ability to maintain osmotic balance via osmolyte accumulation with minimum energy consumption.

In previous studies, sodium accumulation and its compartmentalization into the vacuoles under moderate salt stress were found to modulate osmotic potential and to help ensure the absorption of water. Together, increased water content and the accumulation of sodium ions help improve the degree of succulence ^32-34^. In this study, *H. caspica* plants maintained a steady WC and accumulated sodium ions at moderate salt concentrations (100-200 mM), resulting in maximal succulence of the assimilating branches—a classic characteristic of halophytes. Shoot succulence results in an increased vacuolar volume for sodium dilution and OA ^35, 36^, which is also essential for the salt tolerance of *H. caspica*.

To maintain an osmotic potential for water uptake under saline conditions, halophytes typically accumulate inorganic ions in the vacuoles. Na^+^ and Cl^−^ are metabolically effective osmotica because the energetic cost of absorbing inorganic ions is far lower than that of organic osmolyte synthesis ^37^. This appears to be the case for *H. caspica*, as the plants accumulated Na^+^ and Cl^−^ under salt stress. Furthermore, the accumulation of these inorganic ions increased with increasing NaCl concentrations. Thus, Na^+^ and Cl^−^ were the main inorganic osmolytes in the vacuoles under salt stress and were concentrated to a much greater extent in the assimilating branches and reproductive organs compared to other organs (Figs. 6 and 7). Table 1 shows that, relative to the total solute concentration, the OA contribution of Na^+^ and Cl^−^ was 39% and 46%, respectively, in the assimilating branches of plants treated with 200 mM NaCl. Thus, OA in *H. caspica* appears to be largely accomplished through the accumulation of Na^+^ and Cl^−^ (Table 1).

Maintaining low Na^+^ and high K^+^ in the cytoplasm is essential for a number of enzymatic activities ^38^. Na^+^ enters plant cells principally through K^+^ transport pathways ^39^. The similarity of Na^+^ and K^+^ in terms of their hydrated ionic radii results in a competition for transport between these two ions at the plasmalemma level, which is the basis of Na^+^ toxicity ^39^. Halophytes under salt-alkaline stress usually absorb Na^+^ and simultaneously diminish the uptake of K^+^ ^37, 40-45^. Moreover, reduced K^+^ content in salt-stressed *H. caspica* plants may be due to a down-regulation of the genes involved in K^+^ transport ^46^. Although the K^+^ content in *H. caspica* assimilating branches did not significantly change with increasing levels of salinity in this study, a decreasing trend in K^+^ content was observed with increasing salt stress. This observation along with the increase in the Na^+^/K^+^ ratio of *H. caspica* under salt stress indicated that salt exposure led to competitive inhibition between Na^+^ and K^+^ absorption as the basis of cytosolic Na^+^ toxicity. Thus, *H. caspica* may have a Na^+^ absorption pathway that is dependent on the K^+^ channel. K^+^ accounted for 8% of the solutes contributing to OA in the assimilating branches of untreated control plants, while the sum of Na^+^ and Cl^−^ was 76% (Table 1). This result underscores the differences between inorganic ions in terms of their OA contribution in the assimilating branches. The relative contribution of K^+^ to the total OA of assimilating branches was reduced from 8% in the control to 1.6% in plants treated with 600 mM NaCl. It is well established that the accumulation of Ca^2+^ is inhibited by salt stress in many plants ^47, 48^. Accordingly, Ca^2+^ accumulation slightly decreased and the Na^+^/Ca^2+^ ratio increased in *H. caspica* assimilating branches in the present study as the salinity levels increased (Fig. 4). These data are consistent with the findings of Chinnusamy et al. (2006)^49^. Although Ca^2+^ levels decreased significantly under salt stress, however, its contribution to OA was minor as a result of the very low proportion of Ca^2+^ to total solutes.

*H. caspica* is a salt-diluted halophyte, and saline ions over-accumulate in its vacuoles under high NaCl levels. Thus, organic solutes that do not interfere with metabolism must be synthesized and further accumulated in the cytoplasm to maintain the water content equilibrium within cells. Previous reports suggest that this represents a common metabolic plant strategy, particularly in halophytes. Betaine and proline are compatible solutes that can be accumulated in response to osmotic stress, and the accumulation of these osmolytes is known to be an important adaptive response to salt and drought stress ^36^. Betaine has long been recognized as a primary osmolyte in plant species belonging to the Chenopodiaceae family ^18^, and its contribution to OA in the cytoplasm was significantly greater than proline and TSS in the present study. Similar findings have been reported in other Chenopodiaceae halophytes, such as *Kochia sieversiana* ^50^, *Suaeda fruticosa* 4*1, Atriplex griffith*^ii 42^, *Salicornia europaea* ^51^ and *Suaeda maritime* ^51^. Increased accumulation of betaine under saline conditions may be a common genetic characteristic of Chenopodiaceae halophytes that underlies their heightened resistance to salt stress. Among the organic solutes investigated, betaine was the only solute that increased dramatically with increasing salinity, and it had the greatest contribution to the OA of *H. caspica* grown in the greenhouse.

Table 1 and Figure 5 clearly show that betaine is the major osmolyte among the three organic solutes investigated under increasing salt stress. With increasing salt stress, the betaine content increased significantly (Fig. 5a), and the percentage of the betaine concentration relative to the total solute concentration also increased (Table 1). At the highest salinity level, the contribution of betaine to the OA of *H. caspica* was 34% of the total of all three organic solutes. Beyond the role of betaine as an efficient osmolyte, betaine is thought to improve tolerance to dehydration ^52^, stabilize the protein structure of the PSII complex, and prevent damage in the cell membranes of salt-stressed plants. The above results indicate that betaine may have an important role in both cellular protection and cytosolic OA in *H. caspica*, regardless of the presence of NaCl.

In general, the accumulation of proline, a major organic osmolyte, is closely related with osmotic stress intensity^53^. In the present study, however, a dramatic accumulation of proline only occurred after the stress intensity passed a certain threshold. For example, at 400 and 600 mM salinity, the proline concentration was increased by 13 and 40 folds compared to that of the control, respectively (Fig. 5b). However, the proline concentration was very low compared to the betaine concentration, and the contribution of proline to OA was insignificant, at 0.01–0.4% of the total solute (Table 1). These findings suggest that the changes in the proline concentration in *H. caspica* may not have resulted from the osmotic stress response.

Under field conditions, the halophytes are well adapted to saline soil and exhibits a high capacity for ion absorption and OA ^36, 54^. In the field investigation of the present study for *H. caspica*, all of the aerial organs of seedlings and mature plants accumulated significantly more Na^+^, Cl^−^ and Mg^2+^ than the roots, with particularly high levels in the assimilating branches and reproductive organs of mature plants. In all organs of *H. caspica* collected from saline soil, the concentration of Na^+^ exceeded that of Cl^−^ (Table 1), indicating a capacity to balance Na^+^ with inorganic anions or organic anions. The accumulation of K^+^ in the reproductive organs was greater than that in other organs of mature *H. caspica*. Additionally, the K^+^ content in seedling roots was greater than that in the assimilating branches. Previous research has established that Ca^2+^ can improve membrane stability under adverse conditions as a membrane-bound molecule, alter the expression pattern of certain genes to induce protein synthesis in response to salt stress, and improve the stress resistance of plants ^4^. Thus, accumulated Ca^2+^ is an important indicator of metabolic regulation for K^+^ uptake and important for maintaining a higher K^+^/Na^+^ ratio. In the present study, the lowest concentrations of Na^+^ and Cl^−^, a relatively high concentration of K^+^, and the highest concentration of Ca^2+^ occurred in the roots. The high Ca^2+^ content in the roots indicated reduced perturbation by toxic ions and might be indicative of a possible role of Ca^2+^ in the regulation of sodium uptake in the roots.

The analysis of organic solutes in various organs of *H. caspica* collected from the saline-alkali saline-alkali soil indicates that the reproductive organs of *H. caspica* grown in saline soil accumulated more betaine, proline and TSS than that in the assimilating branches. In contrast, there was no significant difference in the accumulation of betaine or proline between the two organs in relatively moderate saline-alkali soil. The substantial accumulation of organic solutes in the reproductive organs might imply a greater tendency of *H. caspica* plants to protect the reproductive organs for seed production under saline conditions. In seedlings grown in the saline soil, the accumulation of the investigated organic solutes was higher in the roots than that in the assimilating branches. In particular, the TSS content of the roots was 11.75 times that of the assimilating branches. Thus, TSS might also be the important organic solute for OA of this species in the field.

The OA mechanism is critical for the survival of *H. caspica* in saline soil. In this context, the fundamental features of *H. caspica* are its ability to accumulate Na^+^ and Cl^−^ in the vacuoles and to accumulate a large amount of organic compounds (such as betain, TSS) in the cytoplasm (Table 1).

**Conclusions**

The results of this study reveals that 100 and 200 mM NaCl were more favorable to the growth of *H. caspica* than the control condition (0 mM NaCl). On the other hand, 400-600 mM NaCl adversely affected growth due to the excess concentration of sodium ions in the solution. The findings also demonstrated that the salt stress response of *H. caspica* involves multiple adaptive mechanisms. Under saline conditions, increased succulence of the assimilating branches resulted in sodium dilution. The absorption of Na^+^ and Cl^−^ and the synthesis of compatible solutes (betaine, proline and TSS) were identified as the key steps underlying osmotic adjustment in *H. caspica*. We therefore conclude that the strong resistance of *H. caspica* to salt stress depends on its key physiological characters, namely, osmoregulatory mechanisms of inorganic ions, particularly Na^+^ and Cl^−^, and greater accumulation of organic solutes. Considering the distribution of inorganic ions and organic solutes in the various tissues and organs, *H. caspica* also exhibited effective osmoregulation in terms of substance distribution for protecting plant growth and development under high salt stress.

**Materials and methods**

*Plant material and growth conditions*

The seeds of *H. caspica* were collected from plants grown wildly in the extremely saline-alkali and semi-desert regions located at the edge of Gurbantunggut Desert in Xinjiang, Northwest China. The seeds were sown in a mixed medium containing potting soil, vermiculite and perlite at a ratio of 2:1:1. After 45 days in a greenhouse at 25-28°C under natural light (16 h light/ 8 h dark) conditions, irrigated with distilled water, the seedlings were transferred to pots containing the same medium, with seven seedlings per pot. Two weeks later, the plants were divided into seven groups that were treated with distilled water containing 0, 100, 200, 300, 400, 500 or 600 mM NaCl, respectively. The treatments were performed once per two days. After 30 days of treatment, the impact of salinity treatment was measured and recorded.

To investigate the same physiological parameters in the field condition, we chose two relati-alkali environments for samples collction (87°31’315’’E, 44°29’745’’ N). It appgion possesses exremely saline-alkali soil and the other has relatively moderate saline-alkali soil (see growing environment as FigureS1 and TableS1 ). A explained in Figure S2, different tissues of mature *H. caspica* plants were corrected. Briefly, the reproductive organs and assimilating branches of mature *H. caspica* plants growing in the saline-alkali or mildly saline-alkali soil were collected; the above-ground and underground tissues of the seedlings from the saline-alkali soil were also collected. For all

*Determination of water content and degree of* *succulence*

Samples of assimilating branches (6-8 cm in length from the tip) were briefly washed with distilled water to remove dust on the surface then dried with absorbent paper to remove the surface moisture. These samples were weighed to determine the fresh weight (FW). For determination of the dry weight (DW), the samples were steamed at 105°C for 10 min then dried at 80°C until a constant weight was achieved). The water content (WC) was calculated as follows: (FW-DW)/DW. The degree of succulence of the assimilating branches was measured as FW/DW.

*Histological preparation and observation of H. caspica assimilating branches*

Corresponding parts of the assimilating branches from the different *H. caspica* treatment groups were selected, immersed in fixative for 48 h, dehydrated, dipped in wax and sliced at an optimal thickness for analysis. The tissue slices were subsequently immersed in 48°C water to ensure they were fully flattened. The slices were dried in a 50°C oven then double stained with safranin and solid green. The tissue sections were observed using a Leica Microsystems DM3000 microscope.

*Determination of ion content in different tissues and organs of H. caspica plants*

Approximate 50 mg dry material was digested with 2 ml HNO_3_, followed by dilution of the samples with deionized water to a ﬁnal volume of 25 ml ^55^. The major inorganic ions (Na^+^, K^+^, Mg^2+^ and Ca^2+^) were analyzed using an atomic absorption spectrometer (Hitachi Z2000).

*Proline, betaine and soluble sugar content determination*

The accumulation of osmolytes such as free proline (Pro) was analyzed using the procedure described by Zhao et al. (2009) with some modifications ^56^. Betaine content was measured using the anthrone-ethyl acetate method ^57^, and the total soluble sugar (TSS) content was analyzed using the sulfuric acid-anthrone method ^58^.

*Statistical analysis*

Statistical analysis was performed using GraphPad Prism software, version 5.0. The physiological parameter data for the organic solute content of wild *H. caspica* plants were subjected to analysis of variance (t-test). Differences among the means for the other physiological parameters were analyzed by one-way ANOVA. Comparisons among the mean values for the different treatments were performed using the least significant difference (LSD) test at a confidence level of P<0.05. The results for all of the physiological parameters were expressed as means ± standard deviation (SD).

**References**

1. Munns, R. & Tester, M. Mechanisms of salinity tolerance. *Annu Rev Plant Biol* **59**, 651-681 (2008).

2. Flowers, T. J., Hajibagheri, M. A. & Clipson, N. J. W. Halophytes. *Q Rev Biol* **61**, 313–337 (1986).

3. Bell, H. L. & O'Leary, J. W. Effects of salinity on growth and cation accumulation of *Sporobolus virginicus* (Poaceae). *Am J Bot* **90**, 1416-1424 (2003).

4. Zhao, K. F., Fan, H. & Ungar, I. A. Survey of halophyte species in China. *Plant Sci* **163**, 491–498 (2002).

5. Ben Amor, N., Ben Hamed, K., Debez, A., Grignon, C. & Abdelly, C. Physiological and antioxidant responses of the perennial halophyte *Crithmum maritimum* to salinity. *Plant Sci* **168**, 889-899 (2005).

6. da Silva, E. C., Nogueira, R. J. M. C., de Araújo, F. P., de Melo, N. F. & de Azevedo Neto, A. D. Physiological responses to salt stress in young umbu plants. *Environ Exp Bot* **63**, 147-157 (2008).

7. Zhao, K. F. & Li, F. Z. Halophyte of China. *Beijing: Science Press*, 48-49, 58-59, 173-177 (1999).

8. Mahajan S, Tuteja N. Cold, salinity and drought stresses: An overview. *Arch Biochem Biophys* **444,** 139–158 (2005)

9. Sharp, R. E., Hsiao, T. C. & Silk, W. K. Growth of the maize primary root at low water potentials II. Role of growth and deposition of hexose and potassium in osmotic adjustment. *Plant Physiol* **93**, 1337-1346 (1990).

10. Weretilnyk, E. A. & Hanson, A. D. Molecular cloning of a plant betaine-aldehyde dehydrogenase, an enzyme implicated in adaptation to salinity and drought. *Proc Natl Acad Sci USA* **87**, 2745-2749 (1990).

11. Greenway, H. & Munns, R. Mechanisms of salt tolerance in nonhalophytes. *Annu Rev Plant Physiol* **31**, 149–190 (1980).

12. Pastori, G. M. & Foyer, C. H. Common components, networks, and pathways of cross-tolerance to stress. The central role of "redox" and abscisic acid-mediated controls. *Plant Physiol* **129**, 460-468 (2002).

13. Kishor, P. B. K., Hong, Z.L, Miao, G. H., Hu, C. A. A. & Verma, D. P. S. Overexpression of △^1^-pyrroline-5-carboxylate synthetase increases proline production and confers osmotolerance in transgenic plants. *Plant Physiol* **108**, 1387-1394 (1995).

14. Garg, A. K. *et al*. Trehalose accumulation in rice plants confers high tolerance levels to different abiotic stresses. *Proc Natl Acad Sci USA* **99**, 15898-15903 (2002).

15. Karakas, B., Ozias-Akins, P., Stushnoff, C., Suefferheld, M. & Rieger, M. Salinity and drought tolerance in mannitol-accumulating transgenic tobacco. *Plant Cell Environ* **20**, 609–616 (1997).

16. Taji, T. *et al*. Important roles of drought- and cold-inducible genes for galactinol synthase in stress tolerance in *Arabidopsis thaliana*. *Plant J* **29**, 417-426 (2002).

17. Bressan, R. A. *et al*. Learning from the Arabidopsis experience. The next gene search paradigm. *Plant Physiol* **127**, 1354-1360 (2001).

18. Flowers, T. J. & Colmer, T. D. Salinity tolerance in halophytes. *New Phytol* **179**, 945-963 (2008).

19. Zhu, J. K. Plant salt tolerance. *Trends Plant Sci* **6**, 66-71 (2001).

20. Legocka, J. & Kluk, A. Effect of salt and osmotic stress on changes in polyamine content and arginine decarboxylase activity in *Lupinus luteus* seedlings. *J Plant Physiol* **162**, 662-668 (2005).

21. Zhao, K. F. & Fan, H. Comparative study on osmotica and their contribution to osmotic adjustment in eu-halophytes and recretohalophytes. *Chin J Appl Environ Biol* **6**, 99–105 (2000).

22. Liu, Y. X., Yang, X. L. & Yao, Y. Y. Flora of desert. *Science Press* **1**, 361 (1985).

23. Qi, C. H., Han, N. & Wang, B.S. Effect of different salt treatments on succulence of *Suaeda salsa* seedlings. *Plant J* **22**, 175-182 (2005) (in Chinese).

24. Radić, S., Peharec Štefanić, P., Lepeduš, H., Roje, V. & Pevalek-Kozlina, B. Salt tolerance of *Centaurea ragusina* L. is associated with efficient osmotic adjustment and increased antioxidative capacity. *Environ Exp Bot* **87**, 39-48 (2013).

25. Volkmar, K. M., Hu, Y. & Steppuhn, H. Physiological responses of plants to salinity: a review. *Can J Plant Sci* **78**, 19–27 (1998).

26. Khan, A. G. *et al*. Physical, chemical and biological characterization of a steelworks waste site at Port Kembla, NSW, Australia. *Water Air Soil Poll* **104**, 389–402 (1998).

27. Slama, I. *et al*. Effect of sodium chloride on the response of the halophyte species *Sesuvium portulacastrum* grown in mannitol-induced water stress. *J Plant Res* **120**, 291-299 (2007).

28. Lissner, J., Schierup, H. H., Comın, F. A. & Astorga, V. Effect of climate on the salt tolerance of two *Phragmites australis* populations. I. Growth, inorganic solutes, nitrogen relations and osmoregulation. *Aquat Bot* **64**, 317–333 (1999).

29. Sheng, Y. M., Shi, D. C., Xiao, H. X. & Xu, Y. Effect of mixed salts with various neutral and alkaline on the growth of sunﬂower. *J Chin Northeast Norm Univ* **4**, 65–69 (1999).

30. Song, J., Feng, G., Tian, C. Y. & Zhang, F. S. Osmotic adjustment traits of *Suaeda physophora*, *Haloxylon ammodendron* and *Haloxylon persicum* in field or controlled conditions. *Plant Sci*  **170**, 113-119 (2006).

31. Lokhande, V. H., Nikam, T. D., Patade, V. Y., Ahire M. L. & Suprasanna, P. Effects of optimal and supra-optimal salinity stress on antioxidative defence, osmolytes and in vitro growth responses in *Sesuvium portulacastrum* L.. *Plant Cell Tiss Organ Cult* **104,** 41-49 (2011).

32. Messedi, D., Labidi, N., Grignon, C. & Abdelly, C. Limits imposed by salt to the growth of the halophyte *Sesuvium portulacastrum*. *J Plant Nutr Soil Sci* **167**, 720–725 (2004).

33. Moseki, B. & Buru, J. C. Ionic and water relations of *Sesuvium portulacastrum* (L). *SciRes Essays* **5**, 35–40 (2010).

34. Slama, I., Ghnaya, T., Savoure, A. & Abdelly, C. Combined effects of long-term salinity and soil drying on growth, water relations, nutrient status and proline accumulation of *Sesuvium portulacastrum*. *C R Biol* **331**, 442-451 (2008).

35. Flowers, T. J. & Yeo, A. R. Breeding for salinity resistance in crop plants: where next? . *Aust J Plant Physiol* **22**, 875–884 (1995).

36. Tuteja, N. Mechanisms of high salinity tolerance in plants. *Methods Enzymol* **428**, 419-438 (2007).

37. Munns, R. Comparative physiology of salt and water stress. *Plant Cell Environ* **25**, 239-250 (2002).

38. James, R. A. *et al*. Photosynthetic capacity is related to the cellular and subcellular partitioning of Na^+^, K^+^ and Cl^-^ in salt-affected barley and durum wheat. *Plant Cell Environ* **29**, 2185-2197 (2006).

39. Blumwald, E. Sodium transport and salt tolerance in plants. *Curr Opin Cell Biol* **12**, 431-434 (2000).

40. Bolwell, G. P. & Wojtaszek, P. Mechanisms for the generation of reactive oxygen species in plant defence – a broad perspective. *Physiol Mol Plant P* **51**, 347-366 (1997).

41. Khan, M. A., Ungar, I. A. & Showalter, A. M. The effect of salinity on the growth, water status, and ion content of a leaf succulent perennial halophyte, *Suaeda fruticosa* (L.) Forssk. *J Arid Environ* **45**, 73-84 (2000).

42. Khan, M. A., Ungar, I. A. & Showalter, A. M. Effects of salinity on growth, water relations and ion accumulation of the subtropical perennial halophyte, *Atriplex griffithii* var. *stocksii*. *Ann Bot-London* **85**, 225-232 (2000).

43. Shi, D. C. & Sheng, Y. M. Effect of various salt–alkaline mixed stress conditions on sunflower seedlings and analysis of their stress factors. *Environ Exp Bot* **54**, 8-21 (2005).

44. Shi, D. C & Wang, D. L. Effects of various salt-alkaline mixed stresses on *Aneurolepidium* *chinense* (Trin.) Kitag. *Plant Soil* **271**, 15-26 (2005).

45. Short, D. C. & Colmer, T. D. Salt tolerance in the halophyte *Halosarcia pergranulata* subsp. *pergranulata. Ann Bot-London* **83**, 207–213 (1999).

46. Zhu, J. K. Regulation of ion homeostasis under salt stress. *Curr Opin Plant Biol* **6**, 441-445 (2003).

47. Benlloch, M., Ojeda, M. A., Ramos, J. & Rodriguez-Navarro, A. Salt sensitivity and low discrimination between potassium and sodium in bean plants. *Plant Soil* **166**, 117–123 (1994).

48. Khan, M. A., Ungar, I. A. & Showalter, A. M. Effects of salinity on growth, ion content, and osmotic relations in *Halopyrum mucronatum* (L.) Stapf. *J Plant Nutr* **22**, 191-204 (1999).

49. Chinnusamy, V., Zhu, J. H. & Zhu, J. K. Salt stress signaling and mechanisms of plant salt tolerance. *Genet Eng (N Y)* **27**, 141-177 (2006).

50. Yang, C. *et al*. Osmotic adjustment and ion balance traits of an alkali resistant halophyte *Kochia sieversiana* during adaptation to salt and alkali conditions. *Plant Soil* **294**, 263-276 (2007).

51. Moghaieb, R. E. A., Saneoka, H. & Fujita, K. Effect of salinity on osmotic adjustment, glycinebetaine accumulation and the betaine aldehyde dehydrogenase gene expression in two halophytic plants, *Salicornia europaea* and *Suaeda maritima*. *Plant Sci* **166**, 1345-1349 (2004).

52. Sakamoto, A. & Murata, N. The role of glycine betaine in the protection of plants from stress: clues from transgenic plants. *Plant Cell Environ* **25**, 163-171 (2002).

53. Hong, Z. L, Lakkineni, K., Zhang, Z. M. & Verma, P. S. Removal of feedback inhibition of δ^1^ -pyrroline-5-carboxylate synthetase results in increased proline accumulation and protection of plants from osmotic stress. *Plant Physiol* **122**, 1129-1136 (2000).

54. Polijakoff-Mayber, A. & Gale, J. Plants in saline environments. *Handbook Plant Crop Physiol* **15** (1975).

55. Nikolic, N., Kostic, L., Djordjevic, A. & Nikolic, M. Phosphorus deficiency is the major limiting factor for wheat on alluvium polluted by the copper mine pyrite tailings: a black box approach. *Plant Soil* **339**, 485-498 (2010).

56. Zhao, X., Tan, H. J., Liu, Y. B., Li, X. R. & Chen, G. X. Effect of salt stress on growth and osmotic regulation in *Thellungiella* and *Arabidopsis* callus. *Plant Cell Tiss Org Cult* **98**, 97-103 (2009).

57. Grieve, C. M & Grattan, S. R. Rapid assay for determination of water soluble quaternary ammonium compounds. *Plant Soil* **70**, 303-307 (1983).

58. Palma, F., Lluch, C., Iribarne, C., García-Garrido, J. M. & Tejera García, N. A. Combined effect of salicylic acid and salinity on some antioxidant activities, oxidative stress and metabolite accumulation in *Phaseolus vulgaris*. *Plant Growth Regul* **58**, 307-316 (2009).

**Acknowledgments**

The authors wish to thank Dr. Fuchun Zhang, Dr. Ji Ma and Mr. Yonghai Liang for assisting with the experimental suggestion, statistical analysis and technical components of this work. We also greatly appreciate Mr. Kevin Adams for careful proofreading and editing for this manuscript. This research was financially supported by the Educational Natural Science Foundation of Xinjiang Higher University (No. XJEDU2011I02), the National Natural Science Foundation of China (No. 31160186) and the 973 Pre-Research Program of the Ministry of Science and Technology, China (No. 2012CB722204).

**Author contribution**

YZ, LL, RY, XY, and BZ designed the experiments and analyzed the data. YZ and BZ wrote the manuscript text. LL, RY and XY performed the experiments. All authors reviewed the manuscript.

**Competing financial interests**

The author(s) declare no competing financial interests.

**Figure legends:**

**Fig. 1** *Halostachys caspica* growth phenotypes under different NaCl treatments for 70 d.

**Fig. 2** The water content (a) and degree of succulence (b) of the assimilating branches of *Halostachys caspica* seedlings under NaCl stress. Different letters above the bars indicate significantly different means at P<0.05. Data indicate means ± SD (n=4).

**Fig. 3** Cross-sections showing the structural differences between the assimilating branches of *Halostachys caspica* treated with 100 mM NaCl (a) and the control (b). P indicates parenchyma cells.

**Fig. 4** Effect of NaCl on the concentrations of the inorganic ions Na^+^ (a), K^+^ (b) and Ca^2+^ (c) and the Na^+^/K^+^ (d) and Na^+^/Ca^2+^ (e) ratios in *Halostachys caspica* assimilating branches. Plants were treated with different concentrations of NaCl for 30 days. Within each figure panel, different letters above the bars indicate significantly different means at P<0.05. The error bars indicate SD (n=3).

**Fig. 5** Effect of NaCl on the concentrations of the organic solutes betaine (a), proline (b) and TSS (c) in *Halostachys caspica* assimilating branches. Plants were treated with different concentrations of NaCl for 30 days. Within each figure panel, different letters above the bars indicate significantly different means at P<0.05. The error bars indicate SD (n=3).

**Fig. 6** Distribution of inorganic ions in different tissues and organs of wild mature *Halostachys caspica* plants. ROX indicates xylem of reproductive organs, and ABX indicates xylem of assimilating branches. For each inorganic ion, different letters above the bars indicate significantly different means at P<0.05. Data indicate means ± SD, (n=4).

**Fig. 7** Distribution of inorganic ions in assimilating branches and roots of wild *Halostachys caspica* seedlings. * , ** and *** indicate significant differences at P<0.05, P<0.01 and P<0.001, respectively. Data indicate means ± SD (n=4).

**Fig. 8** Distribution of the organic solutes betaine, proline and TSS in different organs of wild mature *Halostachys caspica* plants (a, b and c) and seedlings (d, e and f). MP-SAS indicates mature plants grown in saline and alkali soil, and MP-MSAS indicates mature plants grown in relatively moderate saline- alkali soil. In panels (a, b and c), the grey and white bars represent the content of a given organic solute in the reproductive organs and assimilating branches, respectively. *, ** and *** indicate significant differences at P<0.05, P<0.01 and P<0.001, respectively. Data indicate means ± SD (n=4).

Table 1 Contribution of inorganic ions and organic compounds to the osmotic adjustment of *Halostachys caspica* under different NaCl concentrations.

| NaCl  concentration(mM) | Inorganic ions (%) | | | | Organic compounds (%) | | |
| --- | --- | --- | --- | --- | --- | --- | --- |
|  | Na^+^ | K^+^ | Ca^2+^ | Cl^−^ | Proline | Betaine | TSS |
| ck | 45.6042+7.19^a^ | 8.0857+0.27^a^ | 2.9618+0.06^a^ | 31.912+4.71^b^ | 0.0105+0.003^bc^ | 7.6747+1.75^c^ | 3.0977+1.43^a^ |
| 200 | 39.3048+1.51^ab^ | 3.9834+0.21^b^ | 1.2282+0.27^b^ | 46.313+1.68^a^ | 0.0681+0.019^bc^ | 8.2213+0.51^c^ | 1.6672+0.17^ab^ |
| 400 | 26.1540+0.85^b^ | 1.1805+0.06^c^ | 0.4995+0.03b^c^ | 49.8429+2.05^a^ | 0.1337+0.008^b^ | 19.3621+1.55^b^ | 1.7911+0.32^ab^ |
| 600 | 32.7191+1.71^ab^ | 1.6197+0.11^c^ | 0.7888+0.19^bc^ | 26.7073+1.37^b^ | 0.4058+0.048^a^ | 34.7758+1.69^a^ | 2.3422+0.05^b^ |

The content of all the solutes in the table were first measured as µmol/g FW, and the data shown above for each treatment group represent the average of three replicates for the ratio of the concentration of a given solute to the total solute concentration. Means within a column followed by the same letter are not significantly different at P<0.05.
