# Supplementary material for: Contribution and distribution of inorganic ions and organic compounds to the osmotic adjustment in Halostachys caspica response to salt stress
Source: Sci Rep. 2015 Sep 9;5:13639. doi: 10.1038/srep13639 (PMC4563356; doi:10.1038/srep13639)
Supplement: Supplementary Materials [file srep13639-s2.pdf]

Supplementary Materials to:

Contribution and distribution of inorganic ions and organic compounds to the osmotic adjustment in *Halostachys caspica* response to salt stress

Youling Zeng [1,*], Ling Li [1], Ruirui Yang [1], Xiaoya Yi [1] & Baohong Zhang [2]

[1] Xinjiang Key Laboratory of Biological Resources and Genetic Engineering, College of Life Science and Technology, Xinjiang University, Urumqi 830046, China, [2] Department of Biology, East Carolina University, Greenville, NC 27858, USA

* To whom correspondence should be addressed. E-mail: zengyouling@xju.edu.cn

Table S1 The analysis report of salt components from extremely saline-alkali soil where *Halostachys caspica* can grow strongly as a reference

Analysis No.	Depth (cm)	pH 1: 5	Conductivity ms/cm	g/kg								
				total salts	CO_3^-	HCO_3^-	Cl^-	SO_4^{2-}	Ca^{2+}	Mg^{2+}	K^+	Na^+
1	0-5	8.96	19.4	90.200	0.014	0.275	1.031	58.797	1.774	0.049	0.162	26.663
2	5-10	8.95	8.58	40.950	0.025	0.165	1.994	25.058	2.204	0.165	0.130	10.404
3	10-15	8.86	9.08	41.750	0.018	0.176	1.117	26.573	1.974	0.049	0.134	11.041
4	15-20	8.87	6.12	28.200	0.029	0.143	1.805	16.978	2.285	0.232	0.112	6.204

Detected by Xinjiang Institute of Ecology and Geography, Chinese Academy of Sciences

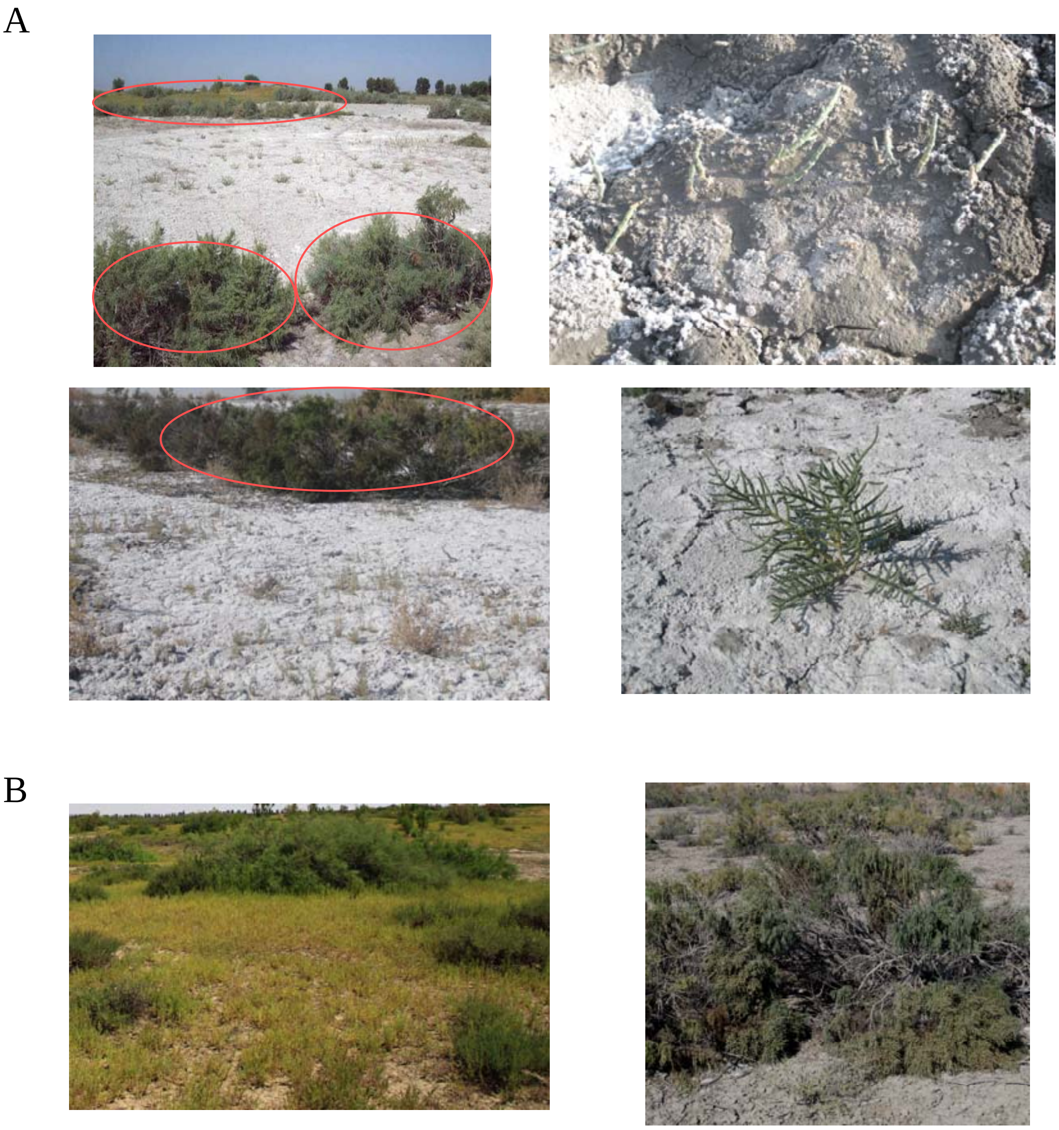

Figure S1 A: *Halostachys caspica* grown in extremely saline-alkali soil in September and October of a year; B: *Halostachys caspica* grown in the relativey moderate saline-alkali soils in June and October of a year.

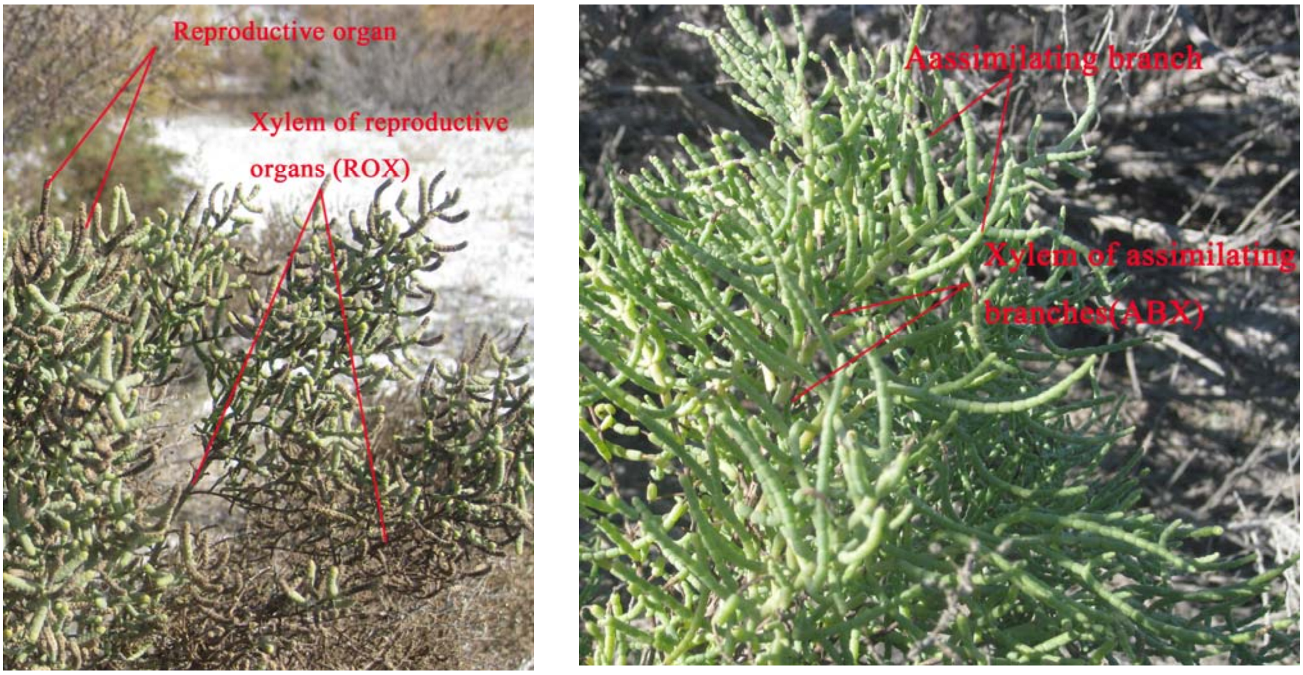

Figure S2 The picture of different tissues position of mature *Halostachys caspica* plants grown in saline-alkali soil in field.
